# The Interplay between Corporate Social Responsibility at Employee Level, Ethical Leadership, Quality of Work Life and Employee Pro-Environmental Behavior: The Case of Healthcare Organizations

**DOI:** 10.3390/ijerph18094521

**Published:** 2021-04-24

**Authors:** Edina Molnár, Asif Mahmood, Naveed Ahmad, Amir Ikram, Shah Ali Murtaza

**Affiliations:** 1 Institute of Management and Organizational Sciences, University of Debrecen, 4032 Debrecen, Hungary; molnar.edina.phd@econ.unideb.hu (E.M.); shah.ali.murtaza@econ.unideb.hu (S.A.M.); 2 Department of Business Studies, Namal Institute, Mianwali 42250, Pakistan; asif.mahmood@namal.edu.pk; 3 Faculty of Management Studies, University of Central Punjab, Lahore 54000, Pakistan; 4 Institute of Business & Management, University of Engineering and Technology, GT Road, Lahore 54890, Pakistan; amir.ikram@uet.edu.pk

**Keywords:** corporate social responsibility, pro-environmental behavior, quality of work-life, ethical leadership, values

## Abstract

The notion of corporate social responsibility (CSR) has been around for many decades. However, even in 2021, its spectrum is still evolving. Several studies addressed CSR for realizing different organizational outcomes. However, its significance in achieving employee-related consequences is relatively new to the literature. In the same manner, it is not clear from existing literature how ethical leaders can impact their followers’ CSR-related behavior, for example, employee pro-environmental behavior (EPB). With this background, the current study aims to explore the relationship of CSR at the employee level (CSR-E) with EPB through the mediating effect of ethical leadership (ELS) in the healthcare sector of a developing economy. This study also proposes a conditional indirect effect of quality of work-life (QWL) in this relationship. The data for the current study were obtained from different hospitals located in a large city through a self-administered questionnaire. The data were examined through the structural equation modeling (SEM) technique. The results validated that CSR-E positively influences EPB, and ELS partially mediates this relationship. Furthermore, the results also confirmed the presence of the conditional indirect effect of QWL in the proposed relationship of the current study. These findings will be helpful for healthcare policymakers to enhance the pro-environmental behavior of employees at the workplace through CSR-E and ELS. These results will also be helpful in reducing the overall environmental footprint of a hospital.

## 1. Introduction

Corporate social responsibility (CSR) is perhaps one of the key concepts in the business environment, and it has dominated almost every sector. Over the years, the phenomenon of CSR has received considerable attention from scholars and practitioners as CSR practices have affected almost every organization in different industries [1]. Until the last decades, the topic of CSR received less attention, but different environment-related issues like climate change [2,3] and global warming [4] forced businesses to adopt sustainability practices to reduce their environmental footprint. Further, “the Paris agreement on climate change” is regarded as a stepping stone toward adapting sustainability practices by global economics [5]. In the recent era, CSR has been regarded as a prerequisite for every sector due to several reasons [6]. CSR is associated with financial improvement [7], organizational reputation [8], operational excellence [9], and organizational commitment [10]. Further, well-established organizations dedicate significant financial resources to CSR activities. However, the success and effectiveness of a CSR strategy depend on the participation of employees, who have a critical strategic role in an organization to attain overall business objectives [11]. It is imperative to empower employees in CSR-related endeavors because without the active participation of internal stakeholders (employees in this case), the organization’s hope to achieve sustainability objectives will remain superficial [12]. The workforce that is skilled and creative is secret to success for an organization, and hence the success of an organization depends on motivating and retaining the skilled workforce [13]. Sen et al. [14] argued that CSR policies and actions of an enterprise have a constructive influence on all major stakeholders, including consumers, employees, and investors.

Different researchers acknowledge the importance of employees to successfully implement various CSR-related programs [15,16,17]. Hence, employee engagement in CSR activities assures that such actions become part of corporate culture and DNA [18]. There is a growing stream of researchers establishing that employees are enactors for an organization to achieve CSR-related outcomes [19,20,21]. However, prior CSR literature in the domain of employees is still limited because most of the previous researchers have explored CSR to achieve other outcomes rather than focusing on employees [4,7,8].

Employee ethical conduct at the workplace has been a central issue in the literature of organizational management. Numerous studies suggest the impetus of employee ethical conduct to achieve different organizational objectives [22,23,24]. With respect to the healthcare sector (the target sector of the current study), scholars have identified different factors that influence the ethical conduct of employees, for example, organizational culture [25], emotional intelligence [26], health consciousness [27], environmental awareness [28], etc. However, an appropriate leadership style is one of the most influential factors that shape employee’s ethical conduct. This is why several researchers have long established that ethical leadership (ELS) positively correlates with employee ethical conduct [29,30,31]. Yet, it is not clear from extant literature how ethical leaders can influence their followers’ CSR-related behavior or, in other words, employee pro-environmental behavior (EPB). Although some researchers explored the relationship of ethical leadership to shape employee pro-environmental behavior [32,33], these studies are inconclusive. Hence, there is a daunting need to do more investigation in this domain. Therefore, the primary purpose of this research enquiry is to test the impact of CSR at the employee level (CSR-E) on EPB in the healthcare sector of Pakistan. The investigation also proposes that ethical leadership mediates this relationship, while the quality of work life (QWL) has a conditional indirect effect on this mediated relationship.

**Figure 1:**
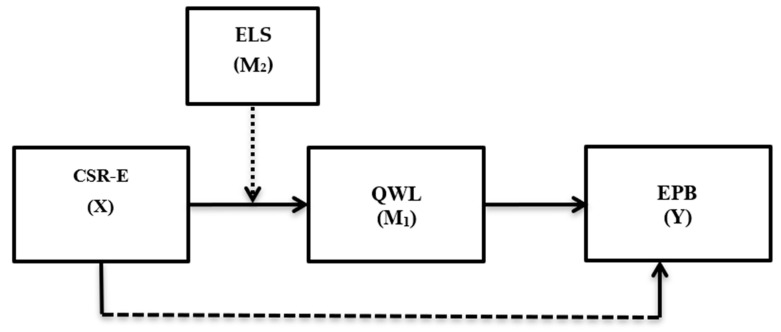
The proposed research model, based on the authors’ conception. This model comprises four variables corporate social responsibility at the employee level (CSR-E) = the independent variable (X), quality of work life (QWL) = the mediating variable (M1), ethical leadership (ELS) = the moderating variable (M2), and employee pro-environmental behavior (EPB) = the dependent variable (Y).

This study intends to test these proposed relationships (Figure 1) in the healthcare sector of Pakistan. This sector is considered relevant for the current study due to three specific reasons. First, the healthcare sector of Pakistan is a labor-intensive sector. However, several researchers have consistently reported that this sector is ignorant in reducing its environmental footprint [34,35,36,37], which is quite distressing for a nation that is already facing extreme climatic conditions, including floods, droughts and extreme temperatures [38,39]. The pandemic of COVID-19 caused a vulnerable situation in this sector as most healthcare workers are in direct exposure to this pandemic as they deal with COVID-19 patients daily. Hence, the employees are expected to consume extra resources (using disposable gloves, masks, dresses) to protect themselves from this pandemic, which also adds to the environmental vulnerability. Second, this sector did not receive due attention from extant CSR researchers, especially how CSR practices of a hospital can help reduce its environmental footprint. Lastly, the healthcare sector is a kind of service sector where several workers perform their jobs round the clock, or in other words, this sector never stops its operations. Hence, addressing CSR-E in this sector is more relevant than other service sectors. Lastly, the healthcare sector of Pakistan primarily deals with the philanthropic orientation of CSR. For example, hospitals are spending their CSR-related funds to treat poor patients free of cost or providing them free medicine, etcetera. However, using CSR to reduce environmental footprint is barely addressed by this sector.

The study contributes to the existing body of literature in a number of ways. First, this study augments concerning the body of existing literature on CSR from the perspective of employees. Prior studies have primarily addressed CSR to achieve other organization-related objectives, including, operational performance [40], organizational efficiency [41], organizational repute [42], organizational commitment [43], corporate performance [44], etc. Moreover, an organization’s CSR engagement is helpful to win the trust of stakeholders even the time of crisis and induces its financial performance [45]. However, addressing CSR at the employee level is a domain of CSR that is still underexplored. Although some research papers signify the importance of CSR at the micro-level [46,47,48,49], these studies are insufficient, etc. This research contends that employees are strategic enablers to reduce the environmental footprint of an organization, and hence, engaging employees in different CSR activities to achieve environmental sustainability is not without logic. Second existing literature is insufficient in clarifying how ethical leaders can influence the pro-environmental behavior of their employees. Although there have been researchers testing the connection of ethical leadership and eco-friendly behavior, most of these investigations were conducted in developed economies [50,51,52], whereas developing countries did not receive due attention in this context. This study argues that developed and developing countries are not alike as they differ in terms of resources, organizational structures, policies, capabilities, and so on. Hence, generalizing the results of developed economies on developing countries is not without potential risks. Third, the mediating effect of ethical leadership between CSR-E and pro-environmental behavior is not well-explored in previous CSR literature from the context of emerging economies. Lastly, employees from the healthcare sector devote considerable time of the lives at workplaces on daily basis. Hence, their work life quality is likely to produce a conditional indirect effect between the mediated relationship of CSR-E, ethical leadership, and environmental-friendly actions. Hence, the introduction of quality of work life as a moderator also adds significantly to the existing literature.

The rest of the research study is comprised of the following parts. The coming part deals with the literature review and theoretical framework. Next comes the methodology part, which deals with sampling procedure, instrument, and data collection approach. After this, there is the data analysis part of the current study in which different statistical tools are applied to get the empirical findings. The last section of this research deals with the discussion and implications part.

## 2. Theory and Hypotheses

The current study receives support from social learning theory [53], the theory of norm reciprocity [54], and means-end chain theory [55] in framing hypotheses. Social learning theory states that individuals learn social behaviors by noticing the behaviors of others. To this end, the current study argues that the ethical behavior of a leader helps shape employee pro-environmental behavior. Because, through the process of role-modeling, when employees see their leader is involved in ethical conduct, they also learn this behavior on their part, and hence they are expected to demonstrate eco-friendly behavior at the workplace [56]. Likewise, the theory of norm reciprocity states that individuals are expected to reciprocate positively for a benefit received from others. In this connection, the current study contends that when employees at the workplace observe that their enterprise is pursuing CSR motives to better society and the environment, they feel positive and want to reciprocate their organization positively. Hence, they are expected to support their organization by performing discretionary or extra-roles, such as pro-environmental behavior. Different researchers have utilized the theory of norm reciprocity for explaining the pro-environmental behavior of employees [17,57,58]. The means-end chain theory argues that individual beliefs are prevailing contrivances that affect the behavior of individuals while they make specific decisions. In this regard, employees receive support from their beliefs and values while they judge the conduct of their organization [59].

CSR refers to the company’s actions to better society beyond what is required by state laws [60]. This investigation is in line with the founding father of CSR, Carroll, in defining CSR as “it is the economic, legal, ethical and philanthropic obligation of a business toward a diverse set of stakeholders” [61,62]. Businesses with strong CSR orientation envisages sustainability and reaps multiple benefits, including high sales [63], positive organizational repute [64], loyal and satisfied employees [65]. Employees are also answerable for the successful operations of an enterprise, including green management, so organizations need to link employee behavior to the organization’s vision [66,67]. Studies have shown that CSR at the employee level is a positive factor in promoting their pro-environmental behavior [17,33,58]. CSR activities at the employee level can help employees shape their eco-initiatives to promote sustainability at all levels of an organization. In agreement with the theory of norm reciprocity, the employees feel optimistic about their organization when they observe that the organization is concerned about influencing society and the environment positively. Hence, according to the theory of norm reciprocity, CSR activities of an organization are positively evaluated by the employees, and in return, they also reciprocate to the organization positively. Thus, the following hypothesis is proposed.

**Hypothesis** **1** **(H1).** *CSR-E of an organization positively relates to employee pro-environmental behavior.*

Prior studies have well emphasized the role of employees in achieving an organization’s CSR objectives [17,19,50]. The literature depicts that institutions can impact their employees’ understanding of CSR-related programs through drafting clear rules and policies [68,69,70]. Now corporate and visionary leaders are persuaded to ensure that their organization adheres to social norms, which require accountability at both the individual and organization levels [71]. Several other scholars have expanded on this argument, suggesting that the social responsibility of an organization should be based on the organizational culture, which is deep-rooted in organizational ethics and norms [72,73,74]. Selecting the right leadership model in an enterprise can increase employee’s involvement in CSR-related tasks [56]. Although leadership is one of the most studied areas in business and management, it is still assumed as an impenetrable area in the literature of organizational management [75,76]. Corporate leaders perform a vital role in engaging their employees in CSR activities [77]. Moreover, personal values and attitudes of leaders toward CSR practices affect the involvement of their organization employees in CSR-related activities [78].

Thus, to successfully achieve CSR-related outcomes, the organizations require ethical leaders who can urge employees to be involved in extra-role or discretionary behaviors [79]. Our study conceptualizes ethical leadership as per the definition of BrownandTreviño [80], who stated that “ethical leaders exert social influence on the followers to promote their ethical conduct. Ethical leaders monitor and promote the good conduct of the organization, leading to creating an ethical character at all levels of the organization [81]. Ethical leaders are considered key personnel in developing and implementing CSR-related tasks within the organization [29]. Hence, ethical leaders set a standard for their employees and can impact employee behavior by building organizational standards and its ethical environment [82]. Therefore, an ethical leader can urge their employees to participate in CSR activities [83]. Ethical leaders and administrators are critical to promoting good values as they support organizations to become more socially responsive. An ethical leader can be CSR-oriented, modeling and promoting the behavior of their employees towards society and the environment at large [58].

Ethical leaders and managers can have enormous financial and non -financial consequences for organizations. One such benefit is motivating employees to demonstrate eco-friendly commitments and actions at the workplace [52]. Ethical leaders emphasize the importance of good morals and socially responsible behaviors to their followers. Thus, in line with social learning theory [53,84], when employees at workplaces observe the ethical conduct of their leaders, it helps them in learning and developing ethics on their part. Further, the social learning process also urges them to demonstrate ethical behavior (pro-environmental behavior) via the role modeling (in this case, ethical leader as a role model) process. Additionally, ethical leaders stress the need for answerability by holding employees liable for their actions. Studies have long established that the process of role-modeling is helpful to alter the behavior of the human capital [85,86,87]. To sum, the authors argue that social learning theory provides ground to the employees to get involved in environment-friendly action and internal corporate social responsibility. Because they take the ethical conduct of their leader as a role model, and hence, they are expected to display pro-environmental behavior. Thus, the following set of hypotheses is proposed.

**Hypothesis** **2** **(H2).** *Ethical leadership positively influences the pro-environmental behavior of employees.*

**Hypothesis** **3** **(H3).** *Ethical leadership mediates between CSR-E and pro-environmental behavior.*

Quality of working life can be defined as “satisfying employees through different needs through resources, actions that come from participation in the workplace” [88]. While serving for an organization, employees typically identify themselves as members of a group with expectations with the organization they work for [89]. The socially responsible behavior of an organization is one of such expectations [90]. An organization that follows CSR principles is likely to make its employees happy with their work because they believe it is responsible for a better work environment for the employees [91]. Moreover, a socially responsible organization makes the employees feel happy about themselves because they believe they are part of a good organization [92].

A competitive market often needs employees to devote many hours to the workplace, and thus job environment becomes an important aspect of the lives of employees. Resultantly, many organizations endeavors to offer methods to provide a better work environment so that their employees have opportunities beyond the workplace, such as ensuring the well-being of employees to improve their lives (quality of work life) [93]. These CSR-related actions can improve employee-workplace relationships to achieve overall organizational growth [94]. In other words, quality of work-life balance focuses on improving employee quality of life at the workplace [95].

On the other hand, CSR is a matter of human capital management because it requires the well-being of the personnel. With all of these in mind, the company’s CSR plans, such as fair compensation/pay, job security, and family support, satisfy its employees and increase employee quality of work life perception [59]. These CSR efforts aim to satisfy the working conditions of employees with respect to healthiness, social life balance and intrinsic factors. Certainly, CSR efforts for employees enhance their quality of life and the organization’s overall performance, including sustainability performance [96]. Hence, as per the means-end chain theory, enlightening employees’ quality of work life makes them committed and enthusiastic. Hence, the human capital willingly serves the organization in achieving its objectives, including sustainability objectives. Therefore, quality of work life is likely to generate a conditional indirect effect between CSR at employee level and pro-environmental conducts. Thus, the following hypothesis is proposed.

**Hypothesis** **4** **(H4).** *Quality of work life moderates the mediated relationship of CSR-E and pro-environmental behavior.*

## 3. Methodology

### 3.1. Sample and Procedure

The healthcare segment of Pakistan was the source of data collection for the current survey. To represent the healthcare sector, the authors purposefully selected five state-of-the-art hospitals from Lahore, the second-largest city in the country. The selected hospitals included Pakistan Kidney and Liver Institute and Research Center (PKLI), Iqraa Medical Complex, Hijaz hospital (HH), Shaikh Zayed Hospital Lahore (SZMC), Ammar Medical Complex Lahore. The explanation for selecting these hospitals lies in the fact that all of these hospitals are vigorously pursuing CSR activities. Likewise, these hospitals are the largest hospitals in the city, where a significant patient load is evident every time. Likewise, these hospital employs thousands of healthcare and administrative workers. The selection of Lahore city lies behind why the city has been consistently receiving a label of “most polluted city” of the world [97]. The city constitutes a multi-million population whose overall health is at stake due to this rising pollution level. Hence, serious measures must be taken at every level to mitigate this widespread level of pollution.

### 3.2. Data Collection Process

Before commencing the actual data analysis process, the spokespersons of the focal hospitals were contacted to seek their support and permission to gather the data from their staff. The authors also signed an undertaking with the ethical bodies of these healthcare institutions to maintain ethical standards in the process of data collection. Further, the authors got informed consent from every respondent to participate in the survey voluntarily. Unfortunately, the widespread COVID-19 pandemic made it impossible for the authors to collect the data from the hospital staff directly. Because the hospital administration of most of the hospitals did not allow the authors to maintain their presence in a hospital for several hours to collect the data, therefore, as an alternative arrangement, the authors, along with the support of spokespersons of the hospitals, asked the hospital administration to arrange for some persons within the hospital, who may collect the data on behalf of the authors. In this regard, the authors requested each hospital to nominate five persons to assemble the data from the sampled hospitals. Thus, the authors provided the necessary training to these persons on how to complete the survey. A total of 1000 surveys were distributed among these five hospitals and received back 489 filled surveys from different respondents, which suggests a healthy response rate of 49% approximately. The data were collected in two stages with a time-lagged difference of 4 weeks. The data collection process was completed during November and December 2020.

### 3.3. Measures

This study adapted the scales from already existing studies, and therefore, the validity and reliability of the survey instrument were pre-tested. To this end, the scale of ethical leadership was adapted from the study of Brown et al. [98], which entailed ten items. In the same way, the scale of CSR-E was adapted from SchaufeliandBakker [99], which comprises three items. Similarly, the three-item scale of environment-friendly actions was adapted from the operationalization instrument of Bissing–Olson et al. [100]. Finally, the authors adapted a nine-item scale of quality of work life from Sirgy, Efraty, SiegelandLee [88]. The authors used a five-point Likert scale for the current survey.

### 3.4. Handling of Social Desirability

In order to address and operationalize the multifaceted issue of social desirability, several measures were undertaken. For example, the survey items were all randomly scattered throughout the questionnaire. The authors did this to break any sequence of answering the responses. This step is also helpful in dealing with the likelihood of any liking and disliking for a particular construct. Likewise, the instrument was checked for accuracy and suitability by experts in the field. This step is necessary to address any ambiguity or confusion in any item statement due to complex or dual-meaning words. Likewise, the authors cleared the data collection team to request the respondents for their true response so that the findings generated by their input may reflect the reality. Table 1 depicts the demographic detail of the sample data.

## 4. Results

### 4.1. Common Method Bias

The authors started the data analysis phase with testing for common method bias (CMB). The CMB test was conducted because the data for all constructs in the current survey was collected from a single respondent. Hence, to doubt the presence of CMB is not without logic. Thus, the authors decided to detect any potential presence of CMB. To this end, a single-factor analysis was undertaken in SPSS as per the recommendation of Harman [101]. In doing so, all the items of the instrument were allowed to be loaded on a single factor. As per the guidelines of Harman, if the output of single-factor analysis validates a single-factor that shares a variance of 50% or more, then it is established that the data calls for some serious attention by the researcher to take care of the issue of CMB. In this regard, the single-factor analysis results confirmed the absence of any such factor that was sharing more than 50% variance. The maximum variance induced by a single factor was 39.68%, which is within the limit of the threshold level. Thus, the authors confirmed that CMB is not a potential concern in the current survey.

### 4.2. Convergent Validity, Factor Loadings, and the Reliability Analyses

In the next stage of data analysis, several tests were deployed to corroborate the reliability and validity of the study. To this end, the authors first tested for convergent validity, which was confirmed through the outcomes of average-variance extracted (AVE) for each construct. For this purpose, the authors assessed the factor loadings of all items of a construct and found no issue in item loadings for a construct because the loading range for all items was beyond the threshold level of 0.5. After verifying the factor loadings, the authors calculated AVE for each variable by taking the sum of squares of all item loadings and then dividing it by the number of items. For example, in CSR-E, there were three items, and hence the authors first calculated the sum of squared loadings of all these three items (CSRE 1, CSRE2, CSRE3) and then dividing it by 3. In this way, the authors calculated AVEs for all constructs. The AVE values provide the base to decide about the establishment of convergent validity because if the value of AVE for a specific construct is higher than 0.5, so it is a confirmation that the concepts of that construct are converging. Hence, the general criterion of convergent validity is established. The consequences of convergent validity (AVE values) for each construct are shown in Table 2. It is observable from the results that all AVEs are beyond the threshold level of 0.5, which means that convergent validity is present in the dataset of the current survey. Likewise, the reliability results were established based on composite reliability (C.R) values. The general rule to establish the reliability of a scale is that the values of CSR should be greater than or equal to 0.7. As per the statistical outcomes reported in Table 2, there is no reliability issue because all four constructs produce sufficient reliability values. Hence, the authors confirmed that there is no issue of reliability in the current survey.

After verifying the validity and reliability results, the authors next performed correlation analysis and discriminant validity analysis as portrayed in Table 3. As per these outcomes, the values of correlation between all constructs are positively significant, which means all the constructs of the current study are positively correlated. As an illustration, the value of the correlation between CSR-E and ethical leadership (ELS) is 0.26^,^ which is positive and significant, validating that these two constructs are positively correlated. For confirming discriminant validity, the authors calculated the square root of AVE for each construct separately. After calculating all square root values of AVEs, the authors compared the square root value of AVE for each construct with correlation values. As per the criterion of FornellandLarcker [102], if correlation values are lesser than square root values of AVE for a construct, the discriminant validity is established. For instance, the square root of AVE for ELS is 0.77, which is larger than the correlation value between CSR-E and ELS (0.26 **). Hence, as per the suggestion of FornellandLarcker [102], the discriminant validity criterion is well-established. The authors also assessed different model fit indices to verify the goodness of data fit. In this regard, the authors observed different model fit values against the standard threshold and found that the results of model fit indices suggested a good fit between theory and data. Lastly, the authors addressed the issue of multicollinearity by checking variation-inflation-factor (VIF). The authors sought support from the guidance of Hair et al. [103] to decide about the presence of multicollinearity in the current survey. As per the criterion of Hair, Black, Babin, AndersonandTatham [103], the overall value of VIF was less than 3, confirming the absence of multicollinearity issue. Hence the authors were confident that there is no probability that multicollinearity can produce any weakening effect of coefficient estimation.

### 4.3. Hypotheses Testing

This study deployed the structural equation modeling technique (SEM) to validate the hypotheses. SEM is a second-generation co-variance-based data analysis technique, which most contemporary scholars prefer to analyze the data at an advanced level [104,105,106] because this technique equips the researchers to estimate different interrelations in a single go. To evaluate the hypotheses of the current study, the authors conducted structural models using AMOS in three ways. In the first place, the authors tested for the direct relations proposed in hypotheses 1 and 2. To this end, the authors executed a structural model without any intervention of mediating or moderating variables. The results of direct effect analysis for hypotheses 1 and 2 are reported in Table 4. According to these results, the model fit values were within the acceptable ranges (*χ*^2^/*df* = 3.26, RMSEA = 0.051, CFI = 0.931, GFI = 0.933, NFI = 0.927). Furthermore, the results of hypothesis 1 were statistically significant (*β1* = 0.34 **, *p* < 0.019), confirming that CSR-E positively influences EPB of the employees in the healthcare sector. Thus, based on these findings, hypothesis 1 is accepted. Likewise, the author verified hypothesis 2 of the current study by repeating the steps mentioned above. In this connection, the results again confirmed that ELS positively relates to EPB, confirming that hypothesis 2 is also significant and true (*β2* = 0.30 **, *p* < 0.05).

In the second place, the structural model was executed to detect the mediating effect of EPB between CSR-E and ELS. To this end, the authors preferred bootstrapping option by choosing a considerably large sample of 2000 via bias-corrected confidence interval with 95%. The approach of bootstrapping to test the mediation effect is preferred by most researchers over the traditional method proposed by BaronandKenny [107]. This traditional approach was heavily criticized by eminent researchers like Hayes [108] and Zhao et al. [109]. Moreover, the Sobel test approach for mediation is also criticized for its inferior power compared to bootstrapping method [110].

The results of bootstrapping approach (Table 5) confirmed that EPB partially mediates between CSR-E and EPB. The authors assumed that there is partial mediation because the beta value was reduced from *β1 =* 0.34 ** to *β3 =* 0.096 **, but still, it is significant (*p* < 0.05). Further, the model fit indices values were also improved than direct effect model meaning that the is an even better fit between theory and data (*χ*^2^/*df* = 2.34, RMSEA = 0.042, CFI = 0.946, GFI = 0.942, NFI = 0.937). Hence, based on these results, hypothesis 3 is accepted, and it is confirmed that ELS mediates between CSR-E and EPB. Lastly, the results of conditional indirect-effect for hypothesis 4 confirmed that there is a conditional indirect-effect of QWL between the indirect connection of CSR-E and EPB (*β4* = 0.116 **, *p* < 0.05).

## 5. Discussion

The current empirical investigation was carried out to test the effect of CSR-E on EPB with this argument that ELS mediates this relationship. Further, the study also proposed a conditional indirect effect of QWL in the mediated relation of CSR-E and EPB. To this end, the results of the current survey validated that CSR-E positively enhances EPB in the healthcare institutions of Pakistan. The respondents of the current survey confirmed that they feel encouraged to practice EPB when observing their organization is concerned with uplifting society and the environment at large through different CSR initiatives. This CSR engagement of their organization plays a key part in aligning their conduct to preserve nature. Hence, they willfully engage themselves in different-extra-role behaviors related to preserve the environment. For example, not switching on the electric lights unnecessarily, using stairs instead of electronic escalators, printing double side of papers, etc. The authors seek support from the theory of norm reciprocity to explain this finding. The theory of norm reciprocity suggests that when persons see CSR commitment of their organization, they feel their organization is benefiting society and nature. Because employees are also part of society, they want to reciprocate their organization’s social initiatives positively. Thus, they stand by their organization to achieve sustainability objectives. The results of the current survey are also endorsed by extant CSR researchers [17,50,66].

Likewise, the current survey results also confirmed the importance of ethical leaders to urge the workplace employees to display discretionary behaviors (pro-environmental behavior in this case). Ethical leaders promote morality at workplaces, and they are expected to set an example for their employees through their ethical conduct. Employees perceive their bosses (ethical bosses) at workplaces as role models, and hence they follow the conduct of their ethical leader. The theory of social learning explains this phenomenon in a better way. According to this theory, the workers are expected to learn ethical behavior after observing their ethical leaders. When they observe that their leader practices ethicality at the workplace, they feel positive to practice such behavior on their part. Hence, this overall process encourages the employees to be involved in environment-friendly actions. Prior literature also supports the notion that ethical leaders help align the environment-friendly deeds of the human capital [31,32,77,86].

In like manner, the current survey results also confirmed the conditional indirect-effect of the superiority of work life between CSR-E and EPB. This result is also logical to explain on significant grounds. For example, the superiority of work life focuses on enhancing the overall working environment for the personnel. When an organization takes serious measures to raise the value of work life of their human capital, it is likely to expect that workers become happy workers who perform their tasks happily. Further, this sense of happiness also urges them to perform an extra role beyond the formal job obligations, such as pro-environmental behavior. Thus, the outcomes of our investigation are consistent with the extract of means-end chain theory in a way that employee views and values affect their overall behavior. Hence, their belief and value system support them in deciding and judging the organizational conduct. This finding also receives support from extant literature [91,94,111]. The organizations following CSR philosophy care for their workers and make every attempt to raise their eminence of work life. Hence, the staff also support such organizations in achieving overall business objectives, including sustainability objectives.

### 5.1. Implications for Theory

This empirical investigation offers significant theoretical and pragmatic contributions. The first theoretical implication of the current study is that it contributes to existing CSR literature from the employee perspectives. Previous studies, however, explored CSR in different contexts [4,7,8] rather than focusing on employees. Further, most of the prior studies on the current topics were conducted in developed economies [78,94]. However, developing economies did not receive due attention. Likewise, prior studies on CSR largely addressed the manufacturing sector, but the phenomenon of CSR in the service sector is not well-explored. Even though there are few studies from the service sector [66,112], the healthcare sector barely received due attention. The study also augments the existing body of knowledge by presenting ethical leadership as a potential mediator, which has given a new aspect to CSR studies to shape the environment-related behavior of their employees by intervening with ethical leaders in this process. Likewise, the conditional indirect effect of superiority of work life is also an interesting addition to the present literature on CSR and employee behavior.

### 5.2. Implications for Practice

The study also offers implications for practitioners and policymakers. The first practical implication of the current study is that it further reveals the importance of CSR-E for healthcare policymakers to achieve their sustainability objectives through employees. Furthermore, the current study also brings it to the surface that through promoting sustainability at the employee level, the country can improve the overall climatic condition, which is a real challenge of the present time. The study also highlights to the policymakers that assuming CSR as a philanthropic phenomenon is an outdated philosophy. In recent times, CSR has gone beyond the philanthropic approach. The country can benefit from the experience of developed countries, especially the countries in the European Union (EU), where a significant improvement in the environment is evident by promoting sustainability practices at all levels. The current state of the healthcare sector in Pakistan is that it spends heavily in the philanthropic domain of CSR by wrongly assuming that this is the whole picture of CSR. This is the time to change this wrong belief and realize that it will not bring any positive hope for the country in the near future without looking CSR to achieve sustainability objectives. Another important practical contribution is that it depicts the significance of ethical leadership in positioning the ethical performance of workers at workplaces. Hence, the healthcare sector policymakers are suggested to promote ethicality among their managers and administrators if they want to reduce their environmental footprint and want to engage their workers in ethical conduct. The policymakers are further suggested to arrange for different training and seminars for their managers and administrators to highlight the importance of their ethical role in promoting sustainability at the employee level. Lastly, it is also imperative for policymakers to realize that improving workers’ quality of work life turns them into happy human capital who support the organization opportunely in achieving its overall objectives.

### 5.3. Limitations and Future Research Directions

Though the research study provides sufficient grounds to accept its theoretical and practical importance, some limitations need to be addressed by future researchers. To this end, the first limitation of the current study originates from explaining employee behavior from the lens of CSR-E. Although the results are significant, caution should be undertaken because human behavior is an intricate phenomenon to comprehend, and there may be some other factors that are important to be considered in the proposed research model. For example, employee perception of life satisfaction, the meaningfulness of work, employee orientation to perform discretionary behavior may be important variables for future researchers to better explain employee behavior. Similarly, the study only considered hospitals that were located in a single city, and hence the geographic concentration raises questions on the generalizability of this research. To address this limitation, future researchers are encouraged to include more cities like Karachi, Faisalabad, Multan, etc. Another constraint of our empirical investigation is that it used cross-sectional data, and predicting causality based on cross-sectional data involves certain risks. Hence, future studies need to employ longitudinal data design. In this research, we utilized a nonprobability sampling approach because, due to some restrictions, the hospitals did not share the list of their employees, and hence the authors were unable to prepare any sampling frame. Thus, like many studies employing a survey methodology, this research is, therefore, not perfectly free from the sampling issue. Future research should use a more systematic approach for data collection to minimize the sampling error. Lastly, in the present research, the proposed theoretical framework and its efficacy were successfully tested in the healthcare sector. For future research, comparing our results with another service sector can be an interesting extension of this research. This theory deepening effort in future research would eventually enhance the value of the proposed theoretical framework.

## Figures and Tables

**Table 1:** Demographic detail.

Demographic	Frequency	%
**Gender**		
Male	233	52.35
Female	256	47.65
**Age group (y)**		
18–25	81	16.56
26–30	117	23.93
31–35	108	22.08
36–40	97	19.84
Above 40	86	17.59
**Experience (y)**		
1–4	89	18.20
5–7	184	37.63
8–10	126	25.77
Above 10	90	18.40
**Total**	**489**	**100**

**Table 2:** Factor loading, convergent validity and reliability results.

Items	Loadings	AVE	C.R
I am passionate about my participation in the company’s CSR	0.76		
Involvement in the company’s CSR motivates me	0.84		
I feel delighted of my engagement in the company’s CSR	0.77	0.63	0.83
My boss attends to employee feedback	0.81		
My boss disciplines workers who disrupt ethical standards	0.84		
My boss conducts his/her personal life in an ethical manner	0.78		
My boss takes care of the interest of employees	0.76		
My boss pursue impartial and balanced decision making	0.74		
My boss can be trusted	0.71		
My supervisor debates business ethics or morals with employees during meetings	0.82		
My boss leads by example of how to ethically do things right	0.69		
My boss explains success not just by outcomes but also by the way that they are attained	0.87		
My boss asks while decision making, “what is the right thing to do?”	0.70	0.60	0.94
I effectively complete the assigned duties in an eco-friendly manner	0.81		
I accomplish tasks specified in my job description in pro-environmental ways	0.72		
I conduct tasks that are assigned to me in an eco-friendly manner	0.72	0.56	0.79
My occupation offers me good health benefits	0.66		
I am content with what I am getting compensated for my work.	0.74		
My profession does well for my family	0.72		
I have good friends at the workplace	0.75		
I have sufficient time to enjoy other things in life	0.68		
I feel respected at work	0.79		
I feel that my profession offers me to realize my full potential	0.73		
My work enables me to improve my professional skills	0.70		
My work allows me to be creative	0.78	0.70	0.91

Notes: loadings = factor loadings, α = Cronbach’s alpha, C.R = composite reliability.

**Table 3:** Correlation, discriminant validity and model fit indices results.

Construct	Mean	SD	CSR-E	ELS	QWL	EPB
**CSR-E**	4.28	0.74	**0.79**	0.26 **	0.29 **	0.36 **
**ELS**	3.97	0.69		**0.77**	0.24 **	0.31 **
**QWL**	4.16	0.78			**0.84**	0.28 **
**EPB**	3.88	0.47				**0.75**
**Model fit indices**	**Range**	**Obtained**				
***χ*^2^/*df***	5.00	3.523				
**RMSEA**	0.08	0.055				
**NFI**	0.90	0.928				
**CFI**	0.90	0.933				
**GFI**	0.90	0.929				

Notes: SD = standard deviation, ** = significant values of correlation, bold diagonal = discriminant validity results.

**Table 4:** The results for hypotheses 1 and 2.

Path	Estimates	S.E	CR	*p*-Value	ULCI	LLCI	Decision
CSR-E → EPB	(*β*1) 0.34 **	0.037	9.19	0.019	0.169	0.428	Accepted
ELS → EPB	(*β2*) 0.30 **	0.037	8.11	***	0.233	0.618	Accepted
Model fit indices	Range	Obtained	*R* ^2^	H1	H2		
*χ*^2^/*df*	5.00	3.26		0.337 *	0.286 *		
RMSEA	0.08	0.049					
NFI	0.90	0.927					
CFI	0.90	0.931					
GFI	0.90	0.933					

Notes: ULCI = upper-limit confidence interval, LLCI = lower-limit confidence interval, **, ***, * = significant values.

**Table 5:** Mediation and moderation results for H3 and H4.

Path	Estimates	SE	Z-Score	*p*-Value	ULCI	LLCI	Decision
CSR-E → ELS→ EPB	(*β3*) 0.096 **	0.027	3.55	***	0.113	0.362	Accepted
CSR-E $\overset{🠇}{\to }$ ELS → EPB	(*β4*) 0.116 **	0.018	6.44	***	0.096	0.231	Accepted
Model fit indices	Range	Obtained	*R* ^2^	H3	H4		
*χ*^2^/*df*	5.00	2.34		0.22 *	0.29 *		
RMSEA	0.08	0.042					
NFI	0.90	0.937					
CFI	0.90	0.946					
GFI	0.90	0.942					

Notes: ULCI = upper-limit confidence interval, LLCI = lower-limit confidence interval, **, ***, * = significant values, SE = standard error.

## Data Availability

The data will be made available on request from the corresponding author.
